# Organic fertilizer substitution in saline-alkali paddy fields: trade-offs among soil quality, greenhouse gas emissions, yield, and profitability

**DOI:** 10.3389/fpls.2026.1882006

**Published:** 2026-07-15

**Authors:** Shuang Liu, Shuoshuo Liang, Zheng Zhao, Wenya Hao, Shuai Gong, Min Zhang, Hua Wang, Qingnan Chu, Rongrong Wang, Huibin Li, Zhimin Sha

**Affiliations:** 1 School of Agronomy, Hebei Agricultural University, Key Laboratory of Crop Growth Regulation of Hebei Province, Baoding, China; 2 School of Agriculture and Biology, Shanghai Jiao Tong University, Shanghai, China; 3 Eco-environmental Protection Institute of Shanghai Academy of Agricultural Sciences, Shanghai, China; 4 Sinochem Modern Agriculture Co., Ltd., Beijing, China; 5 Shanghai Fengxian Agriculture Technology Promotion Center, Shanghai, China; 6 Institute of Agricultural Resources and Environment, Jiangsu Academy of Agricultural Sciences, Nanjing, China

**Keywords:** methane, nitrous oxide, organic fertilizer, profitability, rice yield, saline-alkali paddy fields, soil quality

## Abstract

Balancing crop productivity with greenhouse gas (GHG) mitigation is a major challenge in saline-alkali rice systems. We conducted a three-season field experiment in Shanghai, China, to evaluate four fertilization regimes: no fertilizer (CK), full chemical fertilization (CT), 50% organic substitution (MF), and 100% organic substitution (OF). We quantified methane (CH_4_) and Nitrous oxide (N_2_O) emissions, rice yield, soil properties, soil enzyme activities, soil quality index (SQI), and net environmental and economic benefits (NEEB). Relative to CT, organic substitution consistently lowered N_2_O emissions but increased CH_4_ emissions. Across the study period, MF reduced cumulative N_2_O emissions by 30.50%, maintained rice grain yield, improved soil quality, and increased NEEB by 31.42%. OF further reduced N_2_O emissions by 48.65% but greatly stimulated CH_4_ emissions and caused clear yield and profit penalties. The increase in CH_4_ under organic substitution was associated with higher soil organic carbon and enzyme activity, whereas N_2_O emissions were closely related to soil inorganic N availability. Overall, MF provided the most balanced outcome among productivity, soil improvement, and environmental performance. These findings indicate that partial replacement of chemical fertilizer with organic fertilizer is a promising management strategy for saline-alkali paddy fields, whereas complete substitution is less suitable because the gains in N_2_O mitigation are offset by increased CH_4_ emissions and lower agronomic performance.

## 1 Introduction

Soil salinization is a major threat to agricultural sustainability and food security, particularly in coastal production regions. Global estimates suggest that salinization will affect a substantial proportion of arable land by 2050 (Hassani et al., 2021; Hamoud et al., 2024). This challenge is especially relevant for rice because major rice-growing regions overlap with areas that are increasingly exposed to salinity, intensive irrigation, sea-level rise, and climate change (Zhao et al., 2017; Kontgis et al., 2019). As a staple crop for nearly half of the global population, rice is highly vulnerable to soil salinization, which is currently threatens approximately 160 million hectares of paddy fields worldwide (Darko et al., 2025). Maintaining rice productivity in salt-affected soils while reducing environmental burdens is therefore a major agroecosystem challenge.

To maintain high grain yields, intensive synthetic N fertilizers are routinely applied in traditional rice systems. However, this over-reliance often triggers low N use efficiency and accelerates undesirable environmental discharges, including N_2_O emissions, NH_3_ volatilization, and N leaching and runoff (Zhong et al., 2021; Liang et al., 2026). Rice cultivation is also an important agricultural source of CH_4_ and N_2_O (Qian et al., 2023; Bao et al., 2024; Chu et al., 2025; Lan et al., 2025a), and nitrogen fertilizer can further stimulate CH_4_ production under flooded conditions (IPCC, 2013; Fang, 2024; Liu et al., 2024). Organic fertilizer substitution has therefore received increasing attention as a strategy to improve soil structure, raise soil organic matter, enhance enzyme activity, and promote nutrient cycling (Chu et al., 2016; Zhai et al., 2022; Chu Q. N. et al., 2023). A previous study has demonstrated that partial substitution with straw mulching in rice-wheat rotation significantly increased soil β-glucosidase activity by 113.26% and soil N-acetyl-β-D-glucosidase activity by 45.74% compared to the control treatment (Dai et al., 2021).

However, under conventional farmland soil conditions, there remains no consistent conclusion regarding the environmental effects of organic substitution practices. Previous field studies have demonstrated that organic fertilizer application significantly reduces N_2_O emissions by 75% in double-cropping rice fields (Yu et al., 2021), Meanwhile, other field observations have reported that organic substitution at 13%–71% replacement rates achieves modest N_2_O mitigation, but triggers a substantial 21%–71% increase in CH_4_ emissions (Zhang G. B. et al., 2022). In addition, most previous evaluations have emphasized yield or farm income, while comparatively few studies have integrated environmental costs into economic assessment. The NEEB framework provides a useful way to evaluate these trade-offs by combining agronomic returns with the environmental cost of GHG emissions (Zhang et al., 2018; Bi et al., 2022). This integrated perspective may be particularly important in saline-alkali soils, where constrained mineralization and altered nutrient dynamics could change the response to organic inputs relative to non-saline systems. Yet evidence from saline-alkali paddy fields remains limited. Most existing saline-alkali soil studies rely on laboratory incubation experiments to investigate GHG emission patterns (Wang et al., 2023a, 2023; Zhang et al., 2024), which cannot accurately reflect the real-world dynamics of soil quality and NEEB under actual field production conditions.

To address this gap, we conducted a three-season field experiment in saline-alkali paddy fields in Chongming District, Shanghai. This study aimed to: (1) quantify CH_4_ and N_2_O emission patterns under different organic fertilizer substitution ratios; (2) assess how substitution affected soil quality using a comprehensive soil quality index; and (3) evaluate trade-offs among GHG emissions, yield, and NEEB to identify a practical fertilization strategy for sustainable saline-alkali rice production.

## 2 Materials and methods

### 2.1 Site description

The field experiment was conducted from June 2023 to November 2025 in Chongming District, Shanghai (31°78′ N, 121°25′ E). The site has a subtropical monsoon climate, with mean annual precipitation of 1441.0 mm and mean annual temperature of 16.6 °C (**Supplementary Figure 1**). Before the experiment, baseline soil properties were determined. The soil had an electrical conductivity (EC) of 272.7 μS cm^−1^ and a pH of 7.8, indicating slightly alkaline conditions. Soil organic carbon (SOC) content was 19.78 g kg^−1^, while total N (TN) 1.95 g kg^−1^, total P (TP) 0.23 g kg^−1^, total K (TK) 5.44 g kg^−1^, available P (AP) 68.12 mg kg^−1^, available K (AK) 56.40 mg kg^−1^, nitrate nitrogen (NO_3_^--^N) 3.3 mg kg^−1^, and ammonium nitrogen (NH_4_^+^-N) 7.7 mg kg^−1^. Soil bulk density was measured at 1.38 g cm^−3^.

### 2.2 Experiment design

The field experiment followed a completely randomized design with four treatments, each with three replicates, for a total of 12 plots (5m × 8m). The treatments were: (1) no fertilizer input (CK); (2) full chemical fertilization representing local conventional practice (CT); (3) 50% substitution of chemical fertilizer N with organic fertilizer (MF); and (4) 100% substitution of chemical fertilizer N with organic fertilizer N (OF). The total N application rate in fertilized treatments was 300 kg N ha^-1^. The ratio of basal, tillering, and booting fertilizer was 3:1:1. Basal fertilization included compound fertilizer (N:P_2_O_5_:K_2_O=25:4:6), organic fertilizer (N:P_2_O_5_:K_2_O=2.22:0.79:0.88), calcium superphosphate (16% P_2_O_5_), and potassium sulfate (52% K_2_O). The organic amendment used in this trial consists of thoroughly decomposed straw compost. Urea was applied as N source for tillering and booting fertilization. Detailed fertilizer inputs are provided in **Supplementary Table 1**. Water management followed a mid-season drainage regime, and pest and weed management followed local farmer practice.

### 2.3 Sampling and measurement

#### 2.3.1 Greenhouse gases emission measurements

GHG fluxes were measured using the static chamber method. For gas sampling, a stainless-steel ring-shaped base was permanently installed in the center of each plot, inserted 10 cm deep with its upper rim flush with the soil surface. Samples were taken for 7 consecutive days after each fertilization; otherwise, samples were taken once a month. During the sampling period, a PVC chamber was mounted onto the base. Each chamber (50 cm × 50 cm × 50 cm) was equipped with a thermometer to record air temperature during sampling. An extra extension segment (50 cm × 50 cm × 60 cm) was stacked atop the fixed base collar as rice grew taller, which provided sufficient internal space for aboveground rice biomass, and gas concentrations were determined with a gas chromatograph (Agilent 7890B, Agilent Technologies, USA). CH_4_ and N_2_O fluxes, cumulative emissions, and global warming potential (GWP) were calculated following (Liao et al., 2023; Zhao et al., 2024). The specific calculation formulas are shown in Equations 1–3.

(1) CH_4_ and N_2_O flux calculations.

Gas samples were collected between 09:00 and 11:00 h by placing the static chamber on the base and drawing headspace gas into evacuated vials using the automatic sampling system (**Supplementary Figure 2**).

(1) $$F=\frac{\rho \cdot v}{A}*\frac{dc}{dt}*\frac{273}{273+T}$$

F is the flux of CH_4_ or N_2_O (mg m^-2^ h^-1^); *ρ* is the density under standard conditions (0.714 kg·m^-3^ for CH_4_ and 1.964 kg·m^-3^ for N_2_O); V is the chamber volume (m^3^); A chamber area (m^2^); dc/dt is the rate of concentration change (ppm·h^-1^); and T is the chamber temperature (°C).

(2) Cumulative CH_4_ and N_2_O emissions.

(2) $$cumulative\;emission=\frac{\sum_{i=1}^{n}\left(F_{i}+F_{i+1}\right)}{2}*\left(t_{i+1}-t_{i}\right)*\frac{24}{100}$$

Global Warming Potential (GWP) was calculated as follows.

(3) $$GWP_{CH_{4}+N_{2}O}=28*CH_{4}+265*N_{2}O$$

The constants 24 and 100 are used for time conversion and unit conversion, respectively; the GWP factors for CH_4_ and N_2_O, based on the IPCC AR5, are 28 and 265, respectively, on a 100-year timescale.

#### 2.3.2 Soil sampling and measurement

Soil samples were collected from the 0–20 cm layer using an auger before transplanting and after harvest in each growing season. After thorough mixing, each soil sample was split into two portions. The portion designated for physicochemical analysis was air-dried, ground, and sieved through a 2 mm mesh, whereas the remainder was immediately frozen at -20 °C for subsequent enzyme assays. Soil physicochemical parameters were determined following standard agrochemical methods (Norman et al., 1985). Specifically, TN and TP were quantified via the Kjeldahl digestion and molybdenum blue spectrophotometry methods, respectively. AP was extracted with 0.5 mol L^−1^ NaHCO_3_ and measured colorimetrically. AK was extracted using 1.0 mol L^−1^ ammonium acetate and determined via inductively coupled plasma optical emission spectroscopy (ICP-OES). To assess mineral N, soils were extracted with 2.0 mol L^−1^ KCl, with ${\text{NH}}_{4}^{+}\text{-N}$ and ${\text{NO}}_{3}^{-}\text{-N}$ concentrations subsequently quantified using the indophenol blue method and ultraviolet spectrophotometry, respectively.

Soil enzyme activities, including N-acetyl-β-D-glucosaminidase (NAG) and β-glucosidase (BG), were measured using commercial enzyme assay kits (Suzhou Dream Rhinoceros Biomedical Technology Co., Ltd., China), as described before (Lan et al., 2025b). Fresh soil samples were air-dried naturally, passed through a 60-mesh sieve, and homogenized with reagents. The mixture was centrifuged at 8000×g for 2 min at 25 °C, and the absorbance of the reaction solution was determined at 405 nm. Enzyme activity was expressed as μmol of 4-nitrophenol produced per gram of dry soil per hour, with one unit of enzyme activity defined as the production of 1 μmol 4-nitrophenol g^−1^ soil h^−1^.

#### 2.3.3 Crop yield and components

Rice plants were harvested from a 1 m² in each plot on November 18, 2023, November 14, 2024 and November 21, 2025 to determine the panicle number and grain yields. Final grain yield and 1000-grain weight were measured after sun-drying at 13% moisture content.

### 2.4 Evaluation of soil quality index

The SQI was calculated from soil properties previously identified as informative indicators of soil quality (Li et al., 2015; Andrews et al., 2002; Liang et al., 2023; Chu J. et al., 2023; Wu et al., 2024a). To evaluate the overall impact of organic fertilizer substitution on soil fertility and biochemical functioning in saline-alkali paddy fields, we selected indicators representing soil carbon and nutrient pools (SOC, TN, TP, TK), available nutrient supply (AP, AK, ${\text{NH}}_{4}^{+}\text{-N}$, ${\text{NO}}_{3}^{-}\text{-N}$), and extracellular enzyme activities related to carbon and nitrogen cycling (β-glucosidase [BG] and β-N-acetylglucosaminidase [NAG]), which were directly measured in this study. These variables are sensitive to organic amendment and fertilizer management and provide a multidimensional assessment of changes in soil nutrient status and biochemical processes accompanying substitution treatments. SQI was calculated in two steps:

(1) Indicator standardization.

All soil variables were standardized to dimensionless scores ranging from 0 to 1 using the following equation:

(4) $$SL=\frac{x-L}{H-L}$$

In Equation 4, where χ denotes the observed value of soil indicator, and H and L represent the highest and lowest values, respectively.

(2) SQI integration.

The overall SQI was then calculated using the SQI-area method:

(5) $$SQI_{-area}=0.5*\sum_{1}^{n}*S_{L}^{2}sin\frac{2\pi }{n}$$

In Equation 5, where SQI represents the integrated soil quality index, n is the number of indicators included in the analysis, and S_L_ is the standardized score for the i-th indicator.

### 2.5 Economic and environmental benefit analysis

Economic performance over the three seasons was evaluated using the NEEB framework calculated according to Equations 6–10 (Yin et al., 2025):

(6) NEEB=EP−EC

where NEEB is net environmental and economic benefits (US$ ha^−1^), EP is economic profit (US$ ha^−1^), and EC stands for environmental cost (US$ ha^−1^).

(7) EP=Income−cost

(8) $$Income=Y_{crop}*P_{crop}$$

where income is total revenue, Y_crop_ is crop yield (kg ha^−1^), and P_crop_ is grain price (US$ ha^−1^).

(9) cost=S+F+M+P+L+I

Cost includes expenditure on seed, fertilizer, machinery, pesticides, labor, and indirect inputs, represented by S, F, M, P, L, and I, respectively. Detailed prices are listed in **Supplementary Table 2**.

(10) $$EC=GHGs*Price_{GHGs}$$

Environmental cost was estimated from CH_4_ and N_2_O emissions expressed as CO_2_-equivalents and multiplied by the corresponding social cost of GHG emissions (US$ Mg^−1^ CO_2_-eq). Because carbon prices fluctuate markedly, we used the 2023 average carbon price (64 CNY t^−1^) as the benchmark (Wei et al., 2025), with 100 CNY ≈ 14.14 US$ based on the 2023 average annual exchange rate of the State Administration of Foreign Exchange.

### 2.6 Statistical analysis

All statistical analyses were performed using IBM SPSS Statistics 26.0 (IBM Corp., Armonk, NY, USA). Prior to analysis, data were checked for normality and variance homogeneity (Levene’s test). Treatment effects were examined via one-way ANOVA, with statistically significant differences (*p* < 0.05) identified through Duncan’s multiple range test. Pearson correlation analysis was conducted to examine the relationships among greenhouse gas fluxes, soil physicochemical properties, enzyme activities, and yield components. Figures were prepared in OriginPro 2024 (OriginLab Corp., Northampton, MA, USA). Structural equation modeling (SEM) was developed in SPSS Amos 24.0 (IBM Corp., Armonk, NY, USA). All data in figures and tables are presented as mean ± standard error (n = 3). Significant differences among treatments are denoted by different lowercase letters (*p* < 0.05).

## 3 Results

### 3.1 GHG emissions and GWP

**Figure 1:**
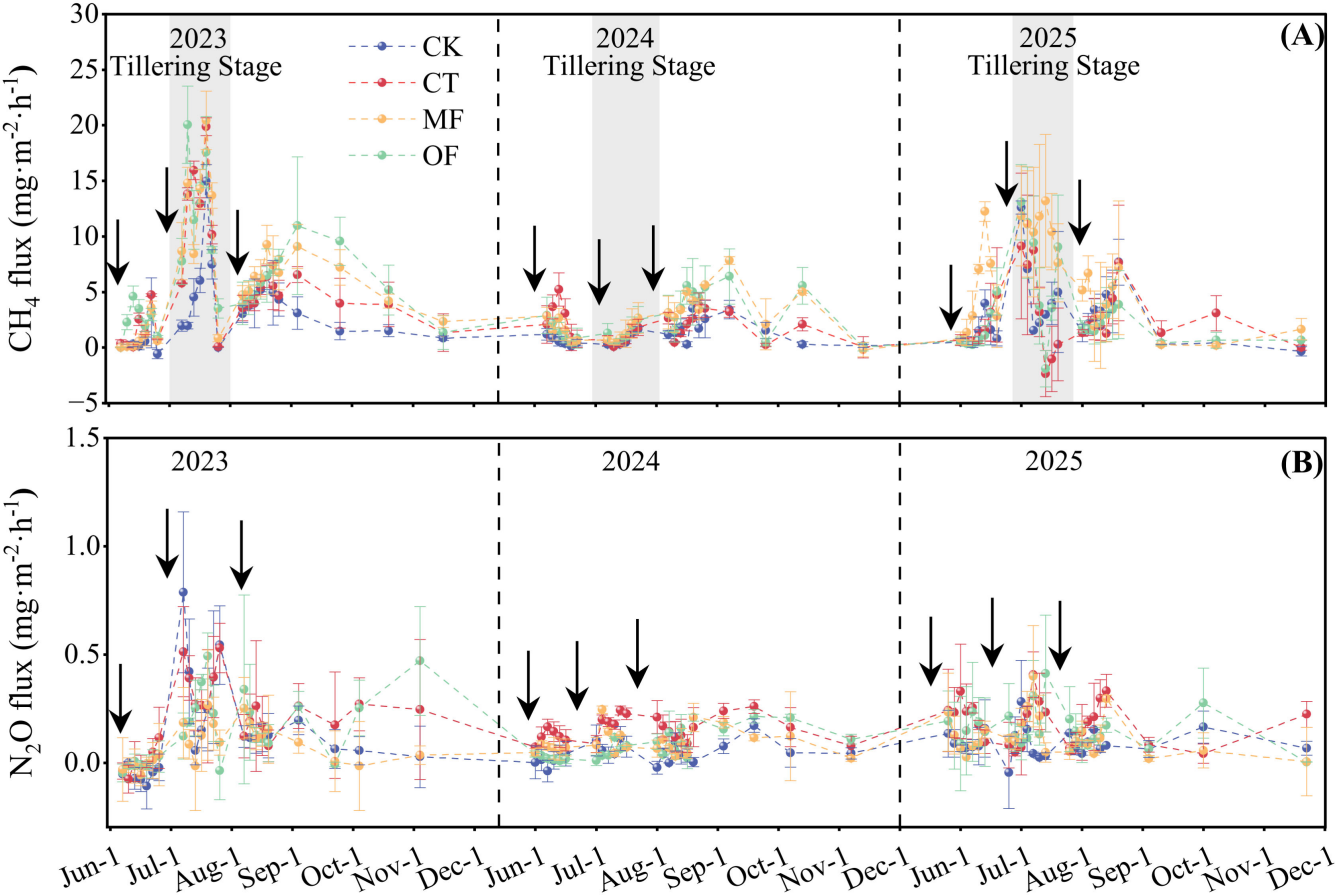
Temporal variation in CH_4_ **(A)** and N_2_O **(B)** fluxes and cumulative seasonal emissions under different fertilization treatments from 2023 to 2025. Black arrows indicate fertilizer application.

CH_4_ and N_2_O fluxes showed clear temporal variations under different fertilization regimes, with CH_4_ emissions typically peaking around the tillering stage. In 2023, peak CH_4_ fluxes occurred after tillering fertilization and reached 20.45 mg·m²·h^−1^ (MF) (**Figure 1A**). Seasonal cumulative CH_4_ emissions ranged from 61.86 kg ha^-1^ in CK to 196.60 kg ha^-1^ in OF (**Supplementary Figure 3A**), and OF emitted 140.46% more CH_4_ than CT (**Supplementary Figure 3A**). In 2024, peak CH_4_ fluxes occurred during booting, with the maximum of 5.56 mg·m²·h^−1^ in OF (**Figure 1A**). Cumulative CH_4_ emissions were lower than in 2023, ranging from 20.06 (CK) to 54.96 (OF) kg·ha^-1^ (**Supplementary Figure 3A**), and OF exceeded CT by 77.39% (**Supplementary Figure 3A**). In 2025, the CH_4_ peak again occurred after tillering fertilization, reaching 13.21 mg m^−^² h^−1^ in MF (**Figure 1A**). Seasonal cumulative CH_4_ emissions ranged from 73.61 to 206.31 kg ha^−1^ (**Supplementary Figure 3A**), and OF exceeded CT by 132.80% (**Supplementary Figure 3A**).

**Figure 2:**
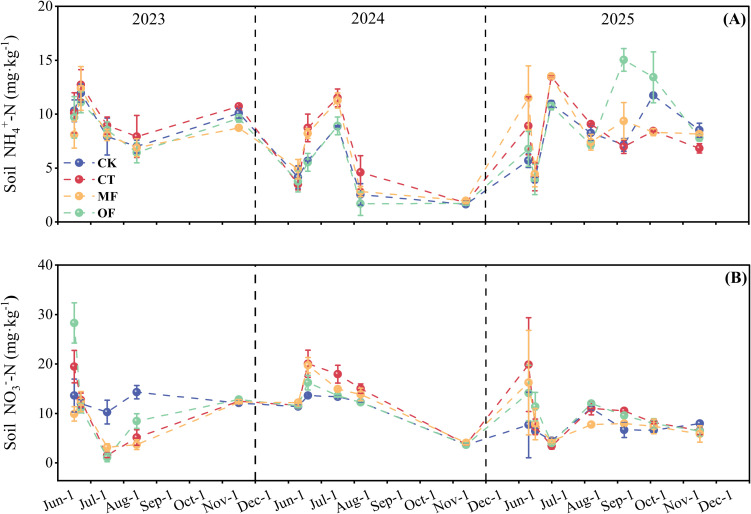
Seasonal patterns of ${\text{NH}}_{4}^{+}\text{-N}$ **(A)** and ${\text{NO}}_{3}^{-}\text{-N}$ **(B)** concentrations in the 0–20 cm soil layer under different fertilization treatments at key rice growth stages during 2023–2025.

N_2_O emissions showed a different response pattern, with strong treatment effects but much lower cumulative emissions than CH_4_. In 2023, cumulative N_2_O emissions ranged from 2.39 kg ha^−1^ in CK to 5.39 kg ha^−1^ in CT (**Supplementary Figure 3B**), and CT emitted 118.22% more N_2_O than OF (**Figure 2B**). In 2024, cumulative N_2_O emissions ranged from 1.44 to 2.84 kg ha^−1^ (**Supplementary Figure 3B**), with CT 76.40% higher than OF (**Supplementary Figure 3B**). In 2025, cumulative N_2_O emissions ranged from 2.33 to 7.31 kg ha^−1^ (**Supplementary Figure 3B**), and CT remained 88.40% higher than OF (**Supplementary Figure 3B**). Overall, organic substitution reduced N_2_O emissions, whereas the increase in CH_4_ became progressively larger with higher substitution rates.

**Figure 3:**
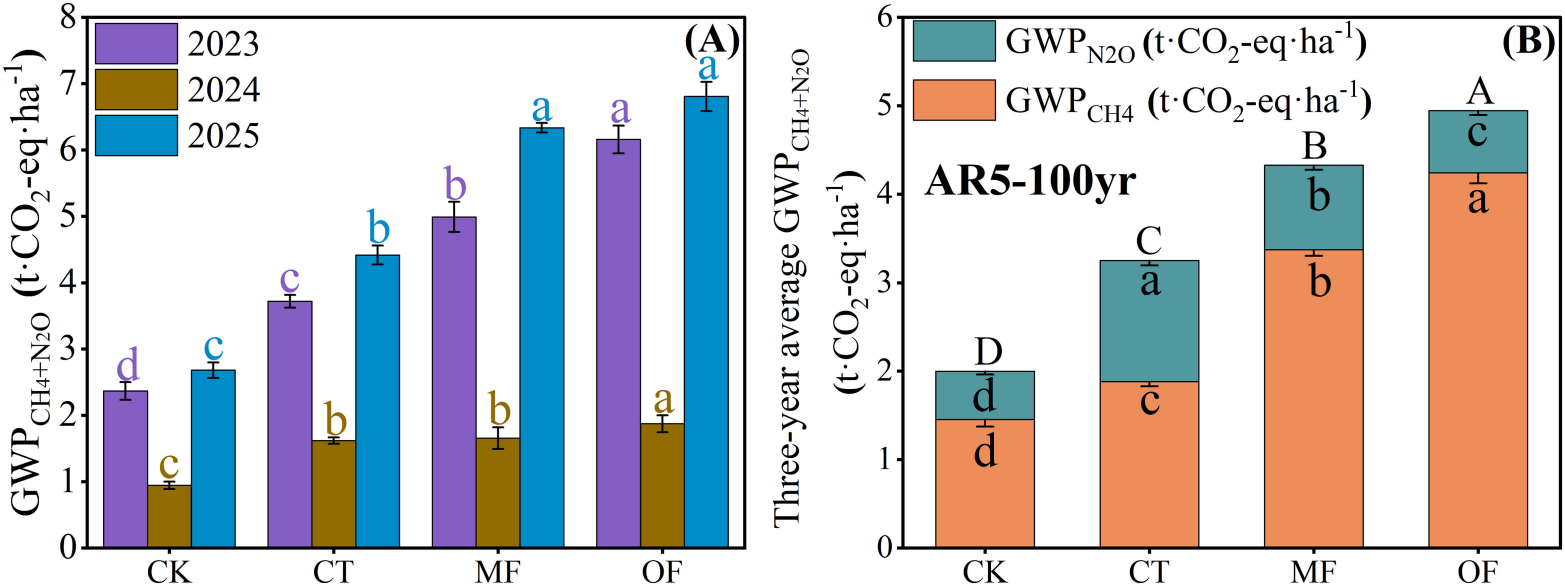
Based on the standards of the IPCC AR5, the GWP **(A)** of different fertilization treatments from 2023 to 2025 on a 100-year timescale. lowercase letters indicate significant differences between treatment groups at the *p* < 0.05 level. Three-year average GWP under different fertilization treatments **(B)**. Lowercase letters indicate significant differences in the GWP of CH_4_ and N_2_O among treatments at the *p* < 0.05 level. Uppercase letters indicate significant differences in the total GWP of CH_4_ and N_2_O among treatments at the *p* < 0.05 level.

Organic fertilizer substitution significantly affected seasonal GWP over the three growing seasons. Compared with CT, OF increased GWP by 65.58% in 2023, 15.78% in 2024, and 54.06% in 2025, whereas MF increased GWP by 30.15% in 2023, and 43.41% in 2025, with no significant difference from CT in 2024 (**Figure 3A**). These results highlight a critical trade-off: although organic fertilizers effectively reduced N_2_O emissions, they simultaneously stimulated CH_4_ production, resulting in a net increase in overall greenhouse gas emissions under OF (**Figure 3B**).

### 3.2 Soil properties

As shown in **Figure 2** and **Supplementary Table 3**, NH_4_^+^-N and NO_3_^--^N displayed clear seasonal dynamics. Soil inorganic N generally increased after fertilization during June–August and then declined toward harvest. Across treatments and years, NH_4_^+^-N peaked between 1.64 and 13.50 mg kg^−1^, whereas NO_3_^−^-N peaked between 1.08 and 28.28 mg kg^−1^ after fertilizer application. Compared with CT, mean NH_4_^+^-N concentrations decreased by 7.22% in MF and 6.54% in OF (**Supplementary Figure 4**). Mean NO_3_^−^-N concentrations decreased by 1.85% in MF and 23.89% in OF. These results indicate that organic substitution reduced soil inorganic N availability, especially soil ${\text{NO}}_{3}^{-}\text{-N}$ under full substitution.

As shown in **Supplementary Figure 5**, from 2023 to 2025, relative to the CT, SOC content increased by 3.95% and 13.01% under the MF and OF treatments, respectively. TN content decreased by 5.68% and 2.16% under MF and OF, respectively. TP content decreased by 0.82% under MF but increased significantly by 109.02% under OF. For TK, MF decreased by 4.28%, whereas OF increased by 1.43%. AP decreased by 2.12% under MF and increased by 1.01% under OF. Similarly, AK decreased by 6.74% under MF and increased by 2.71% under OF.

### 3.3 Soil enzyme activity

**Figure 4:**
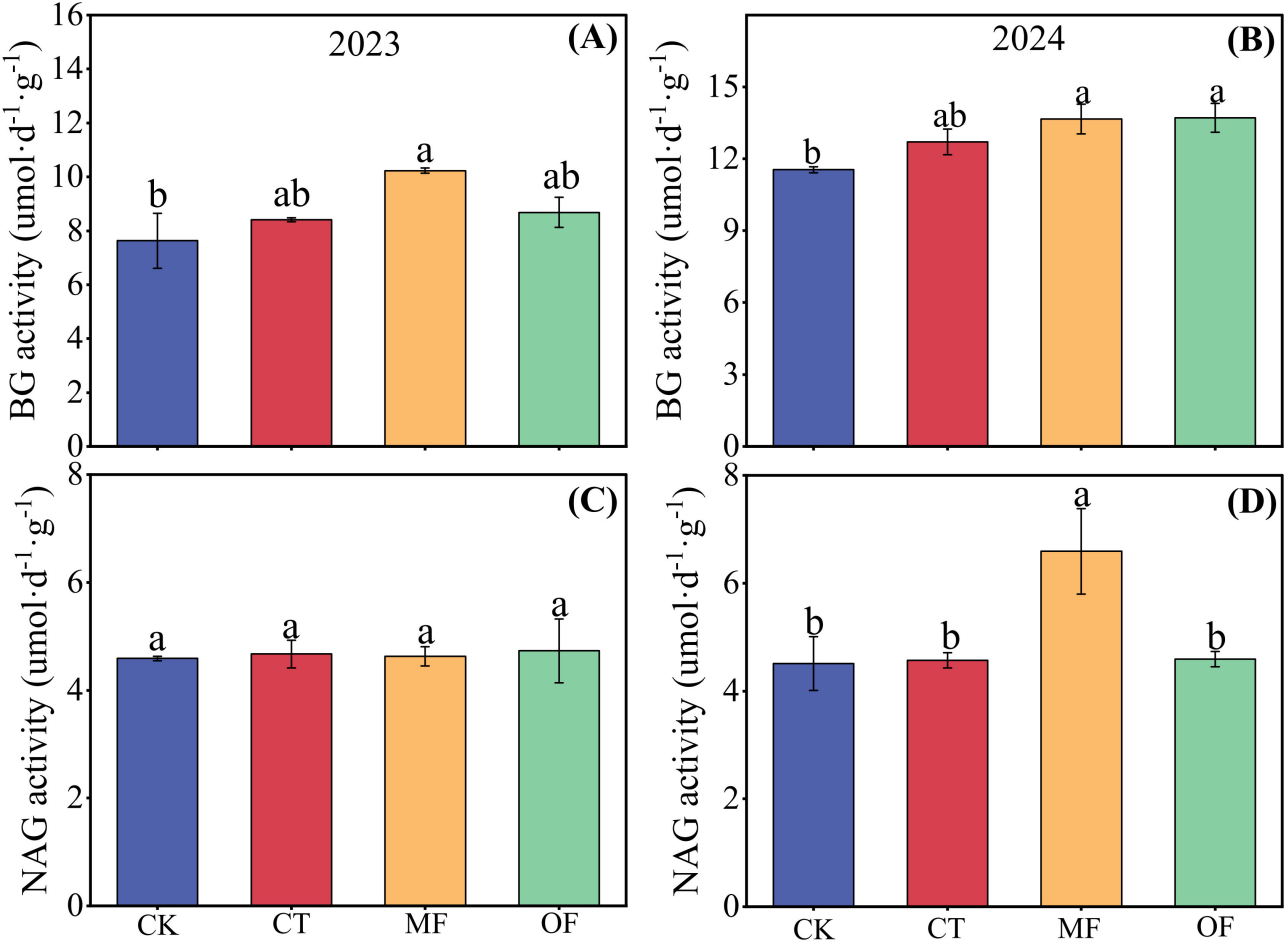
Soil β-glucosidase [BG; **(A, B)**] and N-acetyl-β-D-glucosaminidase [NAG; **(C, D)**] activities in the 0–20 cm soil layer at rice harvest in 2023 and 2024. Different lowercase letters indicate significant differences between treatment groups at the *p* < 0.05 level.

Organic fertilizer substitution significantly enhanced soil enzyme activities related to C and N cycling. In 2023, BG activity in MF was 21.64% higher than in CT, whereas OF did not differ significantly from CT (**Figure 4A**). In 2024, both organic substitution treatments increased BG activity, by 12.24% in MF and 12.57% in OF relative to CT (**Figure 4B**). NAG responded differently. No significant treatment effect was observed in 2023 (**Figure 4C**), but in 2024 the MF treatment increased NAG activity by 44.20% relative to CT (**Figure 4D**). Overall, partial substitution consistently stimulated soil enzyme activity, especially in the later phase of the experiment.

### 3.4 SQI

**Figure 5:**
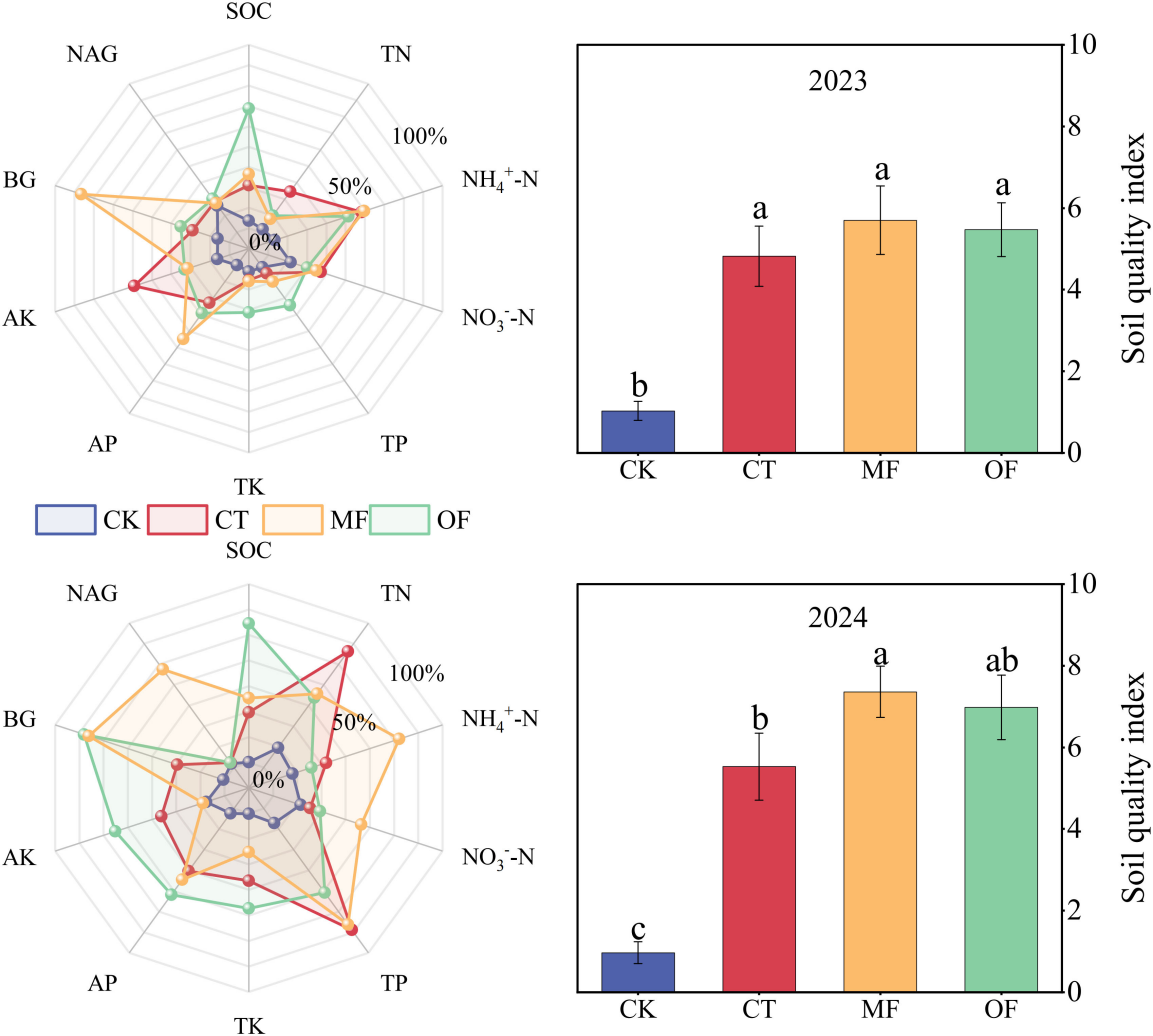
Radar plots of soil physicochemical and biochemical indicators and the corresponding SQI under different fertilization treatments in 2023 and 2024. SOC, soil organic carbon; TN, total nitrogen; TP, total phosphorus; TK, total potassium; AK, available K; AP, available P; ${\text{NH}}_{4}^{+}\text{-N}$, ammonium nitrogen; ${\text{NO}}_{3}^{-}\text{-N}$, nitrate nitrogen; NAG, N-acetyl-β-D-glucosaminidase; BG, β-glucosidase. Different lowercase letters indicate significant differences between treatment groups at the *p* < 0.05 level.

The integrated SQI was significantly affected by fertilization strategy (**Figure 5**). Radar plots showed that most soil quality indicators were higher under organic substitution (MF and OF) than under CK, whereas CT generally had intermediate values. In 2023, all fertilized treatments had significantly higher SQI than CK, but differences among fertilized treatments were not significant. In 2024, the benefits of organic substitution became more pronounced: SQI increased by 33.09% in MF and 26.22% in OF relative to CT (*p<* 0.05). These results indicate that organic inputs improved soil quality, with the clearest advantage observed under partial substitution.

### 3.5 Yield and yield components

**Figure 6:**
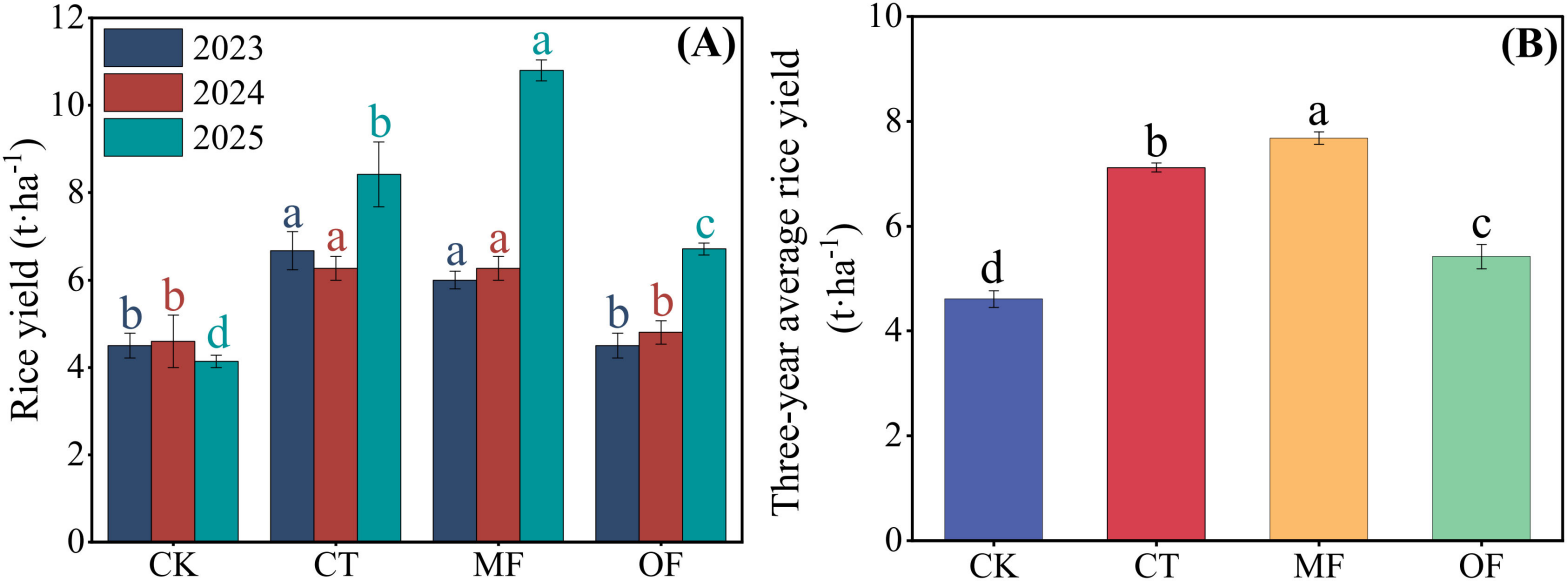
Rice yield under different fertilization treatments from 2023 to 2025 **(A)**, and the three-year average rice yield **(B)**. Different lowercase letters indicate significant differences between treatment groups at the *p* < 0.05 level.

Rice yield differed significantly among fertilization treatments over the three growing seasons (**Figure 6**). OF consistently produced the lowest yield. MF did not differ significantly from CT in 2023 or 2024, and in 2025 the yield under MF was 28.33% higher than under OF (*p<* 0.05). Across the three seasons, MF increased rice yield by 7.87% relative to CT, indicating that partial organic substitution maintained or slightly improved yield under saline-alkali conditions.

Yield component analysis showed that treatment effects were mainly associated with panicle number (**Supplementary Table 4**). CT produced significantly more panicles than OF in all three years and more panicles than MF in 2024 and 2025 (*p* < 0.05). By contrast, grain number per panicle and 1000-grain weight did not differ significantly among fertilization treatments in most cases. These results suggest that yield variation among treatments was driven primarily by differences in panicle formation rather than grain filling.

### 3.6 Mechanism underlying yield formation and GHG emissions under different fertilizer regimes

#### 3.6.1 Correlation analysis

**Figure 7:**
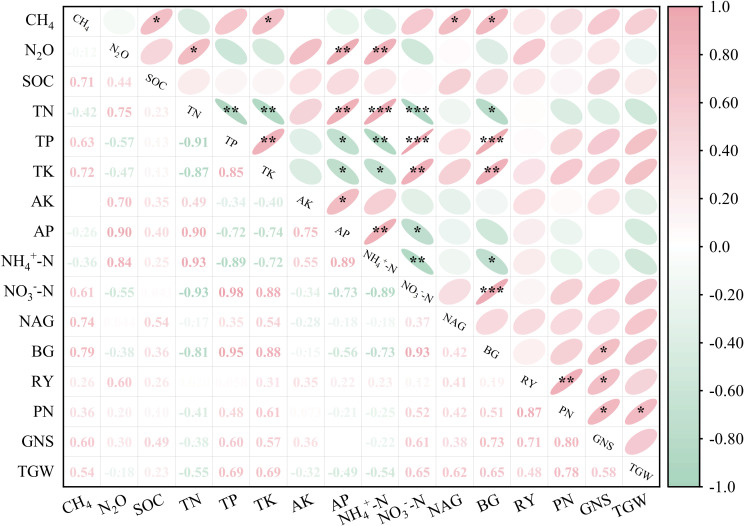
Pearson correlation matrix among greenhouse gas emissions, soil properties, and rice yield components under different fertilization treatments. Pink and green indicate positive and negative correlations, respectively, and ellipses thickness reflects correlation strength. *, **, and *** indicate *p* < 0.05, 0.01, and 0.001, respectively. SOC, soil organic carbon; TN, total N; TP, total P; TK, total K; AK, available K; AP, available P; NH4+-N, ammonium nitrogen; NO3--N nitrate nitrogen; NAG, soil N-acetyl-β-D-glucosidase glycosidase; BG, soil β-glucosidase; RY, rice yield; PN, panicle number; GNS, grain number per spikes; TGW, thousand-grain weight.

Soil CH_4_ emissions were significantly and positively correlated with SOC, TK, NAG, and BG (*p* < 0.05), Soil N_2_O emissions were significantly and positively correlated with TN (*p* < 0.05), AP (*p* < 0.01) and NH_4_^+^-N (*p* < 0.01). Rice yield was significantly and positively correlated with panicle number (PN, *p* < 0.01) and grain number per panicle (GNP, *p* < 0.05), and PN was significantly and positively correlated with rice yield (*p* < 0.01), grain number per spike (*p* < 0.05), and 1000-grain weight (*p* < 0.05) (**Figure 7**).

#### 3.6.2 SEM analysis

**Figure 8:**
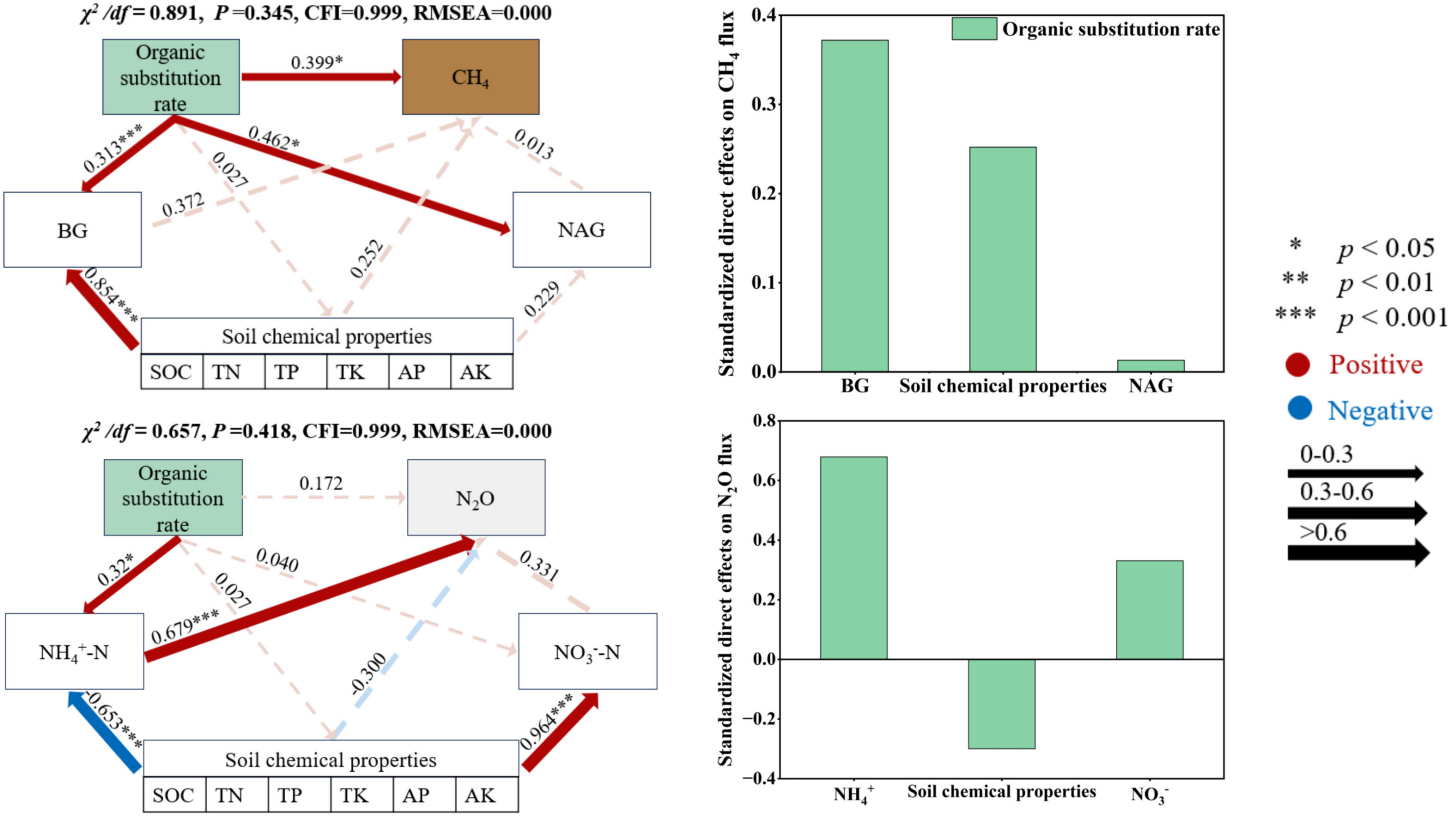
Structural equation models showing the effects of organic fertilizer substitution ratio, soil N availability, enzyme activity, and soil chemical properties on CH_4_ and N_2_O emissions. Numbers beside arrows are standardized path coefficients. Arrow width reflects effect strength. Solid red and blue lines indicate significant positive and negative relationships, respectively; dashed lines indicate non-significant relationships.

As shown in **Figure 8**, SEM was used to disentangle the effects of organic substitution ratio, soil N availability, enzyme activity, and soil chemical properties on CH_4_ and N_2_O emissions. The models showed good fit (CH_4_: *χ*²*/df* = 0.891, *P* = 0.345, CFI = 0.999, RMSEA = 0.000; N_2_O: *χ*²*/df* = 0.657, *P* = 0.418, CFI = 0.999, RMSEA = 0.000). Organic substitution ratio had a significant positive direct effect on CH_4_ emissions (path coefficient = 0.399, *p* < 0.05). BG, soil chemical properties, and NAG also showed positive direct effects on CH_4_, although these were not significant. By contrast, the organic substitution ratio did not directly affect N_2_O emissions. NH_4_^+^-N had a strong positive direct effect on N_2_O emissions (path coefficient = 0.679, *p* < 0.001), whereas the effects of soil chemical properties and NO_3_^−^-N were not significant.

### 3.7 NEEB

**Figure 9:**
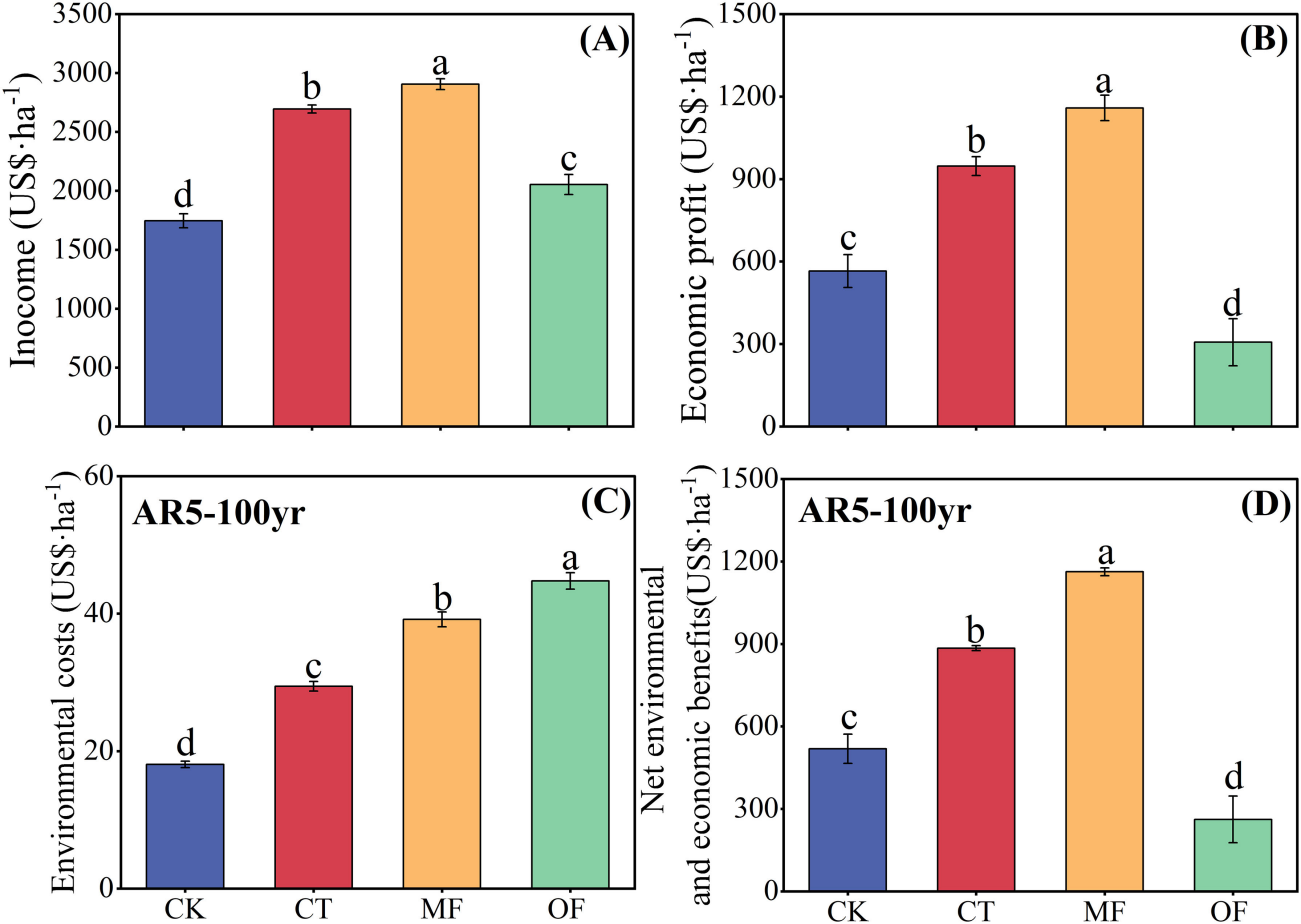
NEEB under different fertilization treatments. Income **(A)**, economic profit **(B)**, Environmental Costs Based on the IPCC AR5 on a 100-Year Time Scale **(C)**, NEEB Based on IPCC AR5 at the 100-Year Time Scale **(D)**. Different lowercase letters indicate significant differences between treatment groups at the *p* < 0.05 level.

Economic analysis showed that MF achieved the highest economic profit, exceeding CT and OF by 22.31% and 278.01%, respectively (**Figure 9**). Environmental cost increased from CK to OF, and was 33.11% higher in MF and 52.14% higher in OF than in CT. When environmental cost was integrated with economic return, MF still produced the highest NEEB, outperforming CT by 31.42% and OF by 344.17%. These results indicate that partial organic substitution provided the most favorable balance between farm profitability and environmental cost.

## 4 Discussion

### 4.1 Organic substitution mitigates N_2_O but exacerbates CH_4_ emissions in saline-alkali paddy fields

Our study revealed a clear trade-off between CH_4_ and N_2_O emissions under organic substitution, which became more pronounced under full substitution. Across the three seasons, organic treatments consistently lowered cumulative N_2_O emissions relative to CT. SEM showed that soil NH_4_^+^-N was the strongest predictor of N_2_O fluxes (standardized path coefficient = 0.679, *p* < 0.001; **Figure 8**), whereas the organic substitution ratio did not exert a significant direct effect on N_2_O. Compared with CT, OF also reduced soil inorganic N availability, particularly NO_3_^−^-N (by 23.89%, **Supplementary Figure 4**). Together with the SEM results, these findings suggest that reduced N_2_O emissions under organic substitution were mainly associated with lower soil inorganic N availability rather than with directly demonstrated changes in nitrification or denitrification. This pattern is consistent with previous studies in saline–alkali soils reporting that limited inorganic N substrates may suppress N_2_O production (Huang et al., 2020; Gao et al., 2023), although the underlying microbial processes were not directly measured in this study.

In contrast, CH_4_ emissions increased with the degree of organic substitution, and SEM identified the substitution ratio as a significant positive direct predictor of CH_4_ fluxes (standardized path coefficient = 0.399, *p* < 0.05; **Figure 8**). Unlike N_2_O, CH_4_ was not directly associated with soil inorganic N in our model, indicating that the two gases responded to organic inputs through different pathways. Pearson correlation analysis further showed that CH_4_ emissions were positively related to SOC and BG activity (**Figure 7**), consistent with the concurrent increases in SOC and enzyme activities observed under organic treatments (**Supplementary Figures S4**, **S5**). In summary, these results suggest that organic substitution may stimulate CH_4_ emissions mainly by increasing carbon substrate availability rather than by altering inorganic N supply. This interpretation is in line with previous studies reporting enhanced CH_4_ release following organic amendment in flooded rice systems, including saline-alkali paddies (Yang et al., 2022; Zhang X. Y. et al., 2022). Persistent flooding during rice cultivation also maintains strongly reducing soil conditions that favor methanogenesis (Liu Q. et al., 2021; Zheng et al., 2024). However, because methanogen abundance, redox potential, and other key biogeochemical variables were not directly measured, these processes cannot be confirmed from our dataset and should be treated as hypotheses for future testing. Targeted microbial and biogeochemical investigations are therefore needed to clarify the controls of CH_4_ emissions under organic substitution in saline-alkali paddy fields.

It should be noted that the climate impact resulting from the trade-off between CH_4_ and N_2_O emissions is influenced by factors such as differences in the assessment time scale, the choice of GWP values, and the selection of emission factors (Neubauer and Megonigal, 2015; Xu et al., 2022). As a short-lived greenhouse gas, the warming effect of CH_4_ on the IPCC 20-year time horizon is significantly higher than its effect on the IPCC 100-year time horizon (**Supplementary Figure 6**), whereas N_2_O, as a long-lived gas, is insensitive to time scales (Ocko et al., 2021; Prather and Wilson, 2026). This implies that organic fertilizer replacement treatments with high CH_4_ emissions will exacerbate the greenhouse effect, particularly on the 20-year timescale. Even on the 100-year timescale, despite the reduced global warming potential of CH_4_, the substantial CH_4_ emissions resulting from organic fertilizer replacement treatments ensure that CH_4_ remains a potentially significant source of GWP (**Figure 3B**). Furthermore, we calculated the global warming potential over 100- and 20-year time scales according to the definitions in the IPCC Sixth Assessment Report and found that methane’s contribution to GWP remains significant (IPCC, 2021). However, the choice of different GWP values has a relatively minor impact on the final results (**Supplementary Figure 6**). This study calculates global warming potential based on field-measured cumulative greenhouse gas emissions; therefore, there is some uncertainty compared to results from studies using non-localized emission factors.

### 4.2 Synergistic effects of partial organic substitution on rice yield and soil quality in saline-alkali paddies

Our results demonstrate that partial organic substitution not only maintained but significantly enhanced rice yield while simultaneously improving the SQI. This synergy aligns with previous findings (Liu B. et al., 2021; Dai et al., 2021), which emphasized the advantages of integrated nutrient management. In the saline-alkali soils, organic amendments play a crucial role in buffering salt stress and ameliorating soil physical structure. By reducing bulk density and increasing porosity, MF facilitates root proliferation and nutrient uptake, thereby mitigating the physiological constraints typically imposed by high salinity (Liu et al., 2026; Du and Yan, 2026).

Notably, while OF improved the SQI (**Figure 5**), it resulted in the lowest grain yield. This phenomenon was mainly due to the asynchrony between nutrient release and crop demand in saline-alkali paddies (Wu et al., 2024b). Excessive organic N supply in OF treatment may lead to insufficient mineral N availability during critical early growth stages due to suppressed mineralization rates under saline stressed conditions. In contrast, MF ensures an immediate supply of mineral N for early growth while leveraging the sustained release of nutrients and biological activation from organic matter for late-season stability (Seufert et al., 2012; Liu J. et al., 2021).

### 4.3 Trade-offs and implications for sustainable rice production

The NEEB analysis conducted in this study indicates that MF is a viable strategy for restoring coastal saline-alkali lands, as it strikes a balance between economic benefits and environmental costs (**Figure 9**). In the management of degraded lands, decision-making often involves trade-offs between reducing greenhouse gas emissions and increasing productivity. Results across different time scales indicate that, under the IPCC’s 20-year GWP accounting framework (short-term scale), the GWP value of CH_4_ is amplified. CH_4_ emissions generated by the use of organic alternatives offset some of the gains from increased yields, ultimately leading to a decline in NEEB. However, the resulting improvements in soil quality and economic benefits far outweigh the negative environmental impacts caused by the increase in GWP (**Supplementary Figure 7**). These findings suggest that the sustainable management of saline-alkali lands should shift from a single-objective approach to a multi-objective optimization framework. Furthermore, given the cumulative and lagged effects of organic amendments on soil remediation, the three-year monitoring period is insufficient to fully evaluate long-term carbon sequestration potential and its intricate coupling with soil desalinization processes.

### 4.4 Study limitations and future research perspectives

Although this three-year field study provides robust evidence for the trade-offs involved in organic fertilizer substitution within saline-alkali paddies, several limitations warrant further exploration. First, the experimental design was restricted to two substitution levels (50% and 100%), among the treatments tested, MF may therefore provide a practical option for reducing chemical fertilizer input and the risk of nitrogen loss while improving soil quality under saline-alkali paddy conditions, although its long-term climate benefits require further verification. future research should incorporate finer gradients to precisely identify the optimal threshold for maximizing NEEB across varying salinity levels. Second, while significant improvements in soil enzymatic activities were observed, the underlying microbial mechanisms—specifically the successional patterns and functional gene abundances of taxa involved in carbon and nitrogen cycling remain to be elucidated through metagenomic or transcriptomic approaches. Furthermore, given the cumulative and lagged effects of organic amendments on soil remediation, the three-year monitoring period is insufficient to fully evaluate long-term carbon sequestration potential and its intricate coupling with soil desalinization processes.

## 5 Conclusions

In saline-alkali paddy systems, organic fertilizer substitution reshapes coupled responses of soil quality, greenhouse gas emissions, rice yield, and economic profitability, indicating that management decisions in degraded coastal paddies should be based on integrated trade-off assessment rather than on a single agronomic or environmental metric. Among the fertilization regimes tested, 50% organic substitution provided the most balanced outcome by reducing N_2_O emissions, improving the soil quality index, maintaining rice yield relative to full chemical fertilization, and achieving the highest net ecosystem economic benefit. By contrast, full organic substitution improved soil quality and N_2_O mitigation but was associated with higher CH_4_-related warming contributions, yield decline, and lower profitability.

Thereafter, we recommend partial organic substitution when the objective is to restore soil fertility while maintaining productivity and economic returns. Full substitution should be applied cautiously, particularly where short-term yield stability and economic performance are important. Policymakers should account for CH_4_ as well as N_2_O when evaluating climate benefits, and should consider amendment type, application timing, and water management to better synchronize nutrient supply with crop demand. Future research should refine substitution rates and organic amendment types to further reduce CH_4_ emissions without compromising the agronomic, soil-quality, and economic advantages observed under MF among the treatments tested in this study.

## Data Availability

The raw data supporting the conclusions of this article will be made available by the authors, without undue reservation.
