# Supplementary material for: Organic fertilizer substitution in saline-alkali paddy fields: trade-offs among soil quality, greenhouse gas emissions, yield, and profitability
Source: Front Plant Sci. 2026 Jul 15;17:1882006. doi: 10.3389/fpls.2026.1882006 (PMC13415759; doi:10.3389/fpls.2026.1882006)
Supplement: Supplementary file 1 [file DataSheet1.docx]

Supplementary Material

## Supplementary Figures


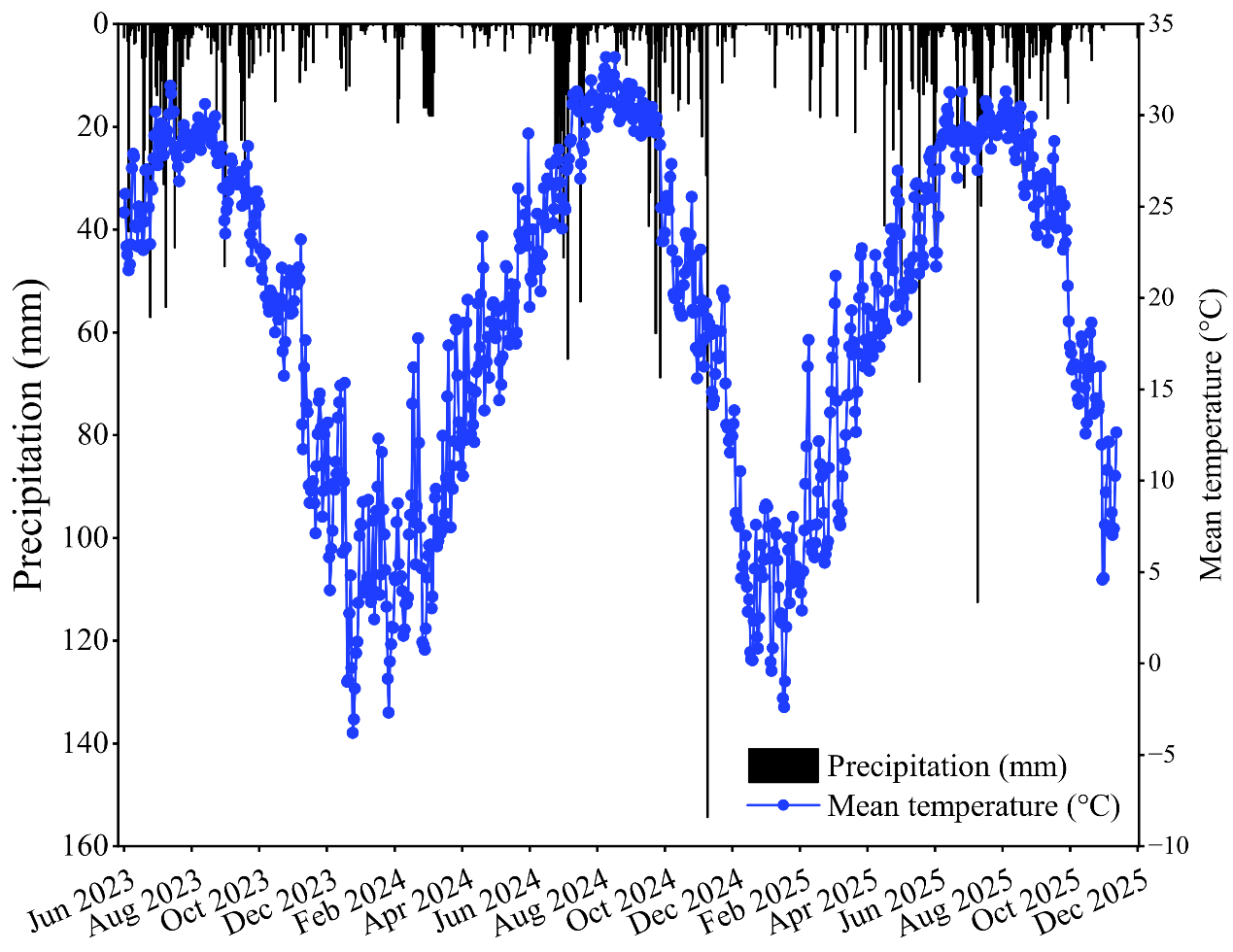


Supplementary Figure 1. Meteorological conditions at the experiment site from June 2023 to December 2025.


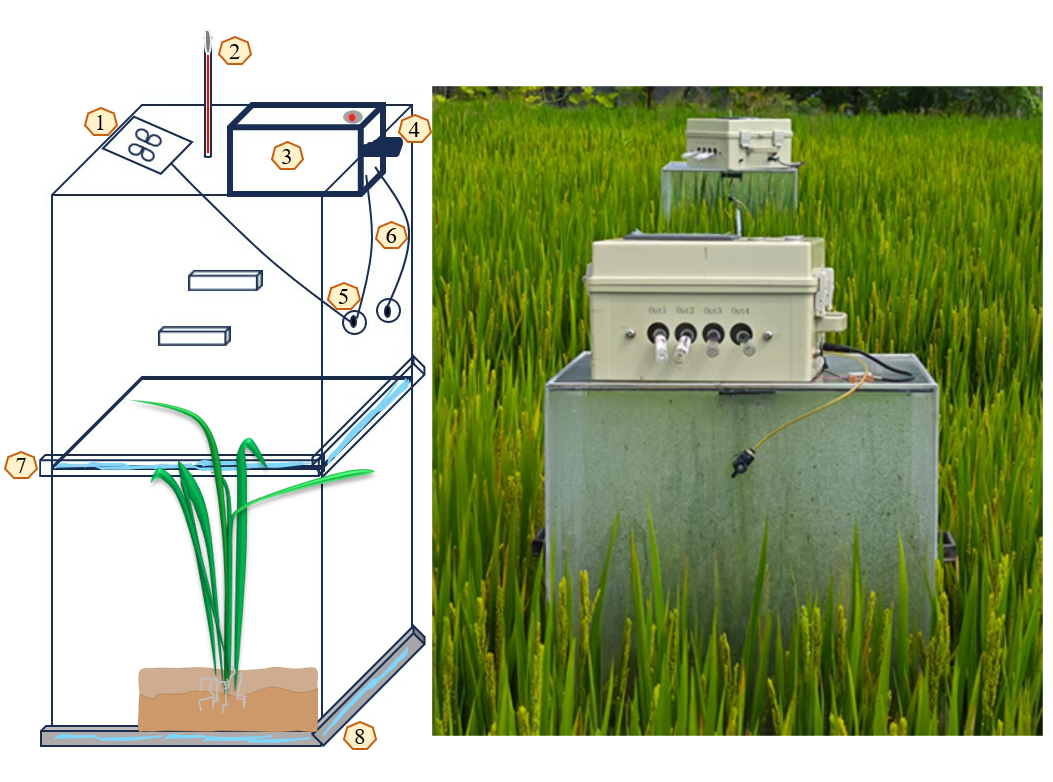


**Supplementary Figure 2.** Schematic diagram of the greenhouse gas sampling system. 1: Fan; 2: Thermometer; 3: Sampling box; 4: Gas cylinder;5: Power cable; 6: Pneumatic horizontal pipe; 7: PVC sink; 8: Square base.

**
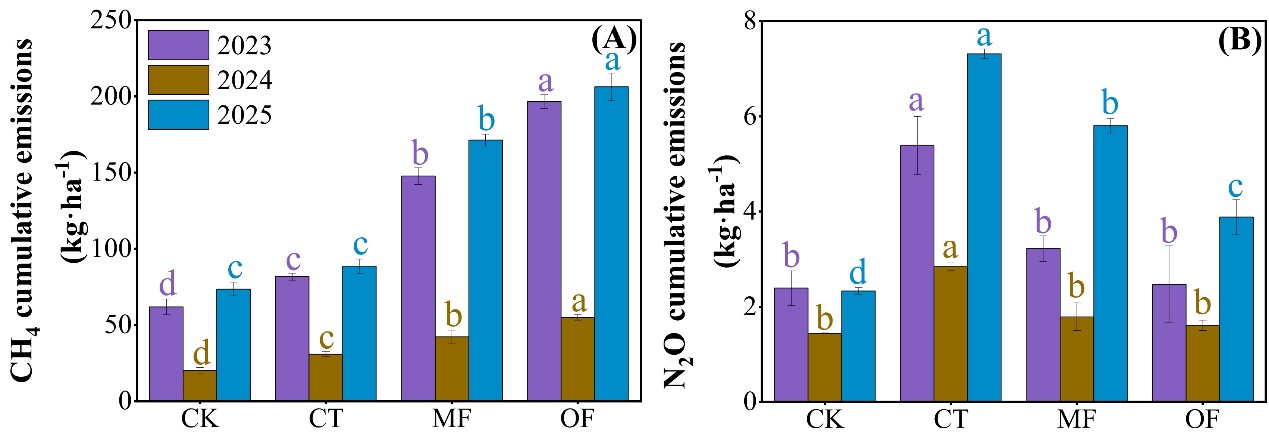
**

**Supplementary Figure 3.** Cumulative emissions of CH₄ (A) and N₂O (B) under different treatments from 2023 to 2025. Different lowercase letters indicate significant differences between treatment groups at the *p* < 0.05 level.

**
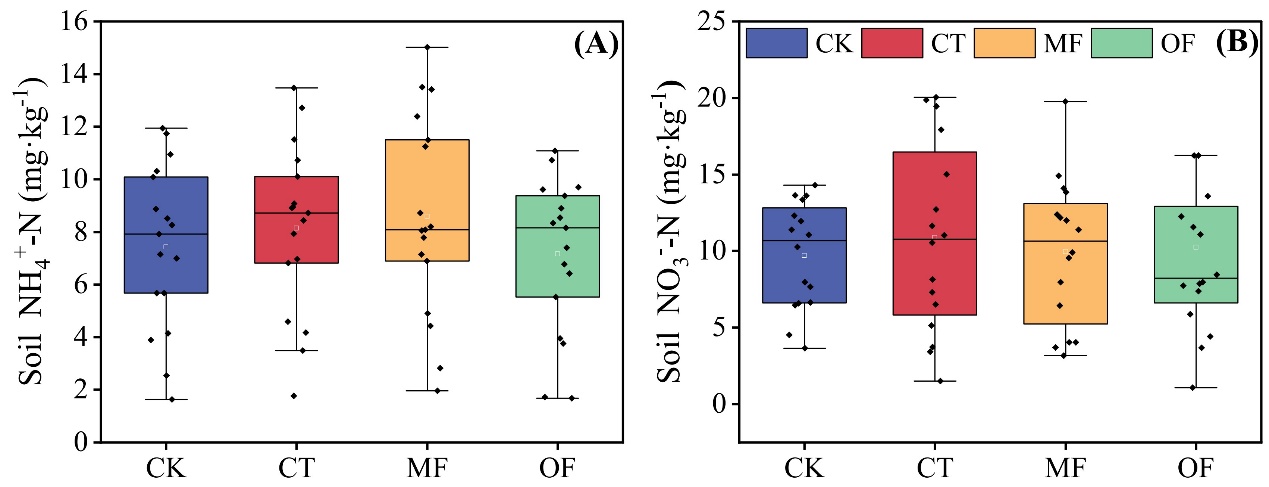
**

**Supplementary Figure 4.** Soil NH₄⁺-N (A) and NO₃⁻-N (B) contents under different fertilization treatments from 2023 to 2025.


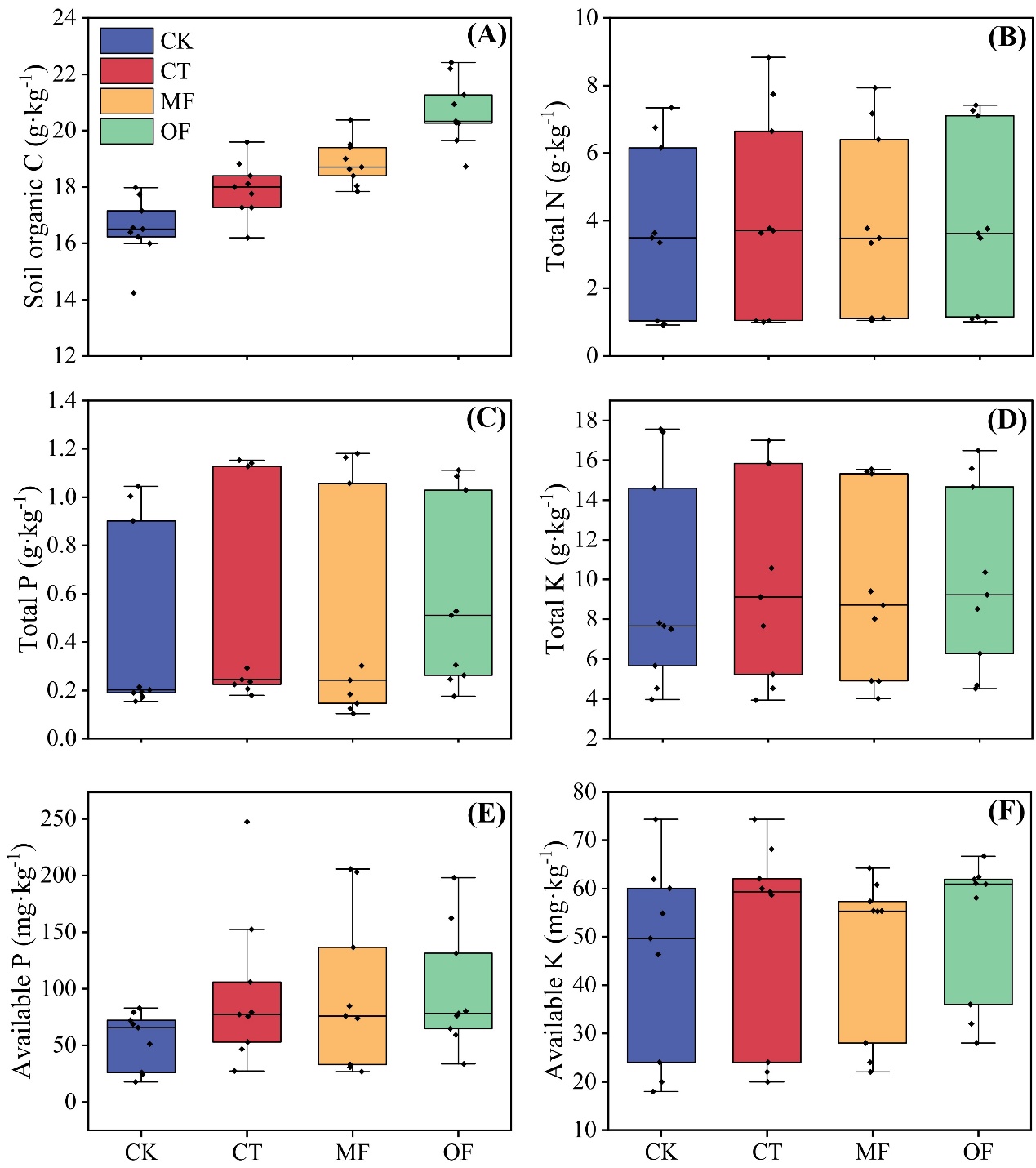


**Supplementary Figure 5.** Soil organic carbon (A), total nitrogen (B), total phosphorus (C), total potassium (D), available P (E), and available K (F) in 0-20 soil layer at rice harvest under different fertilization treatments from 2023 to 2025.


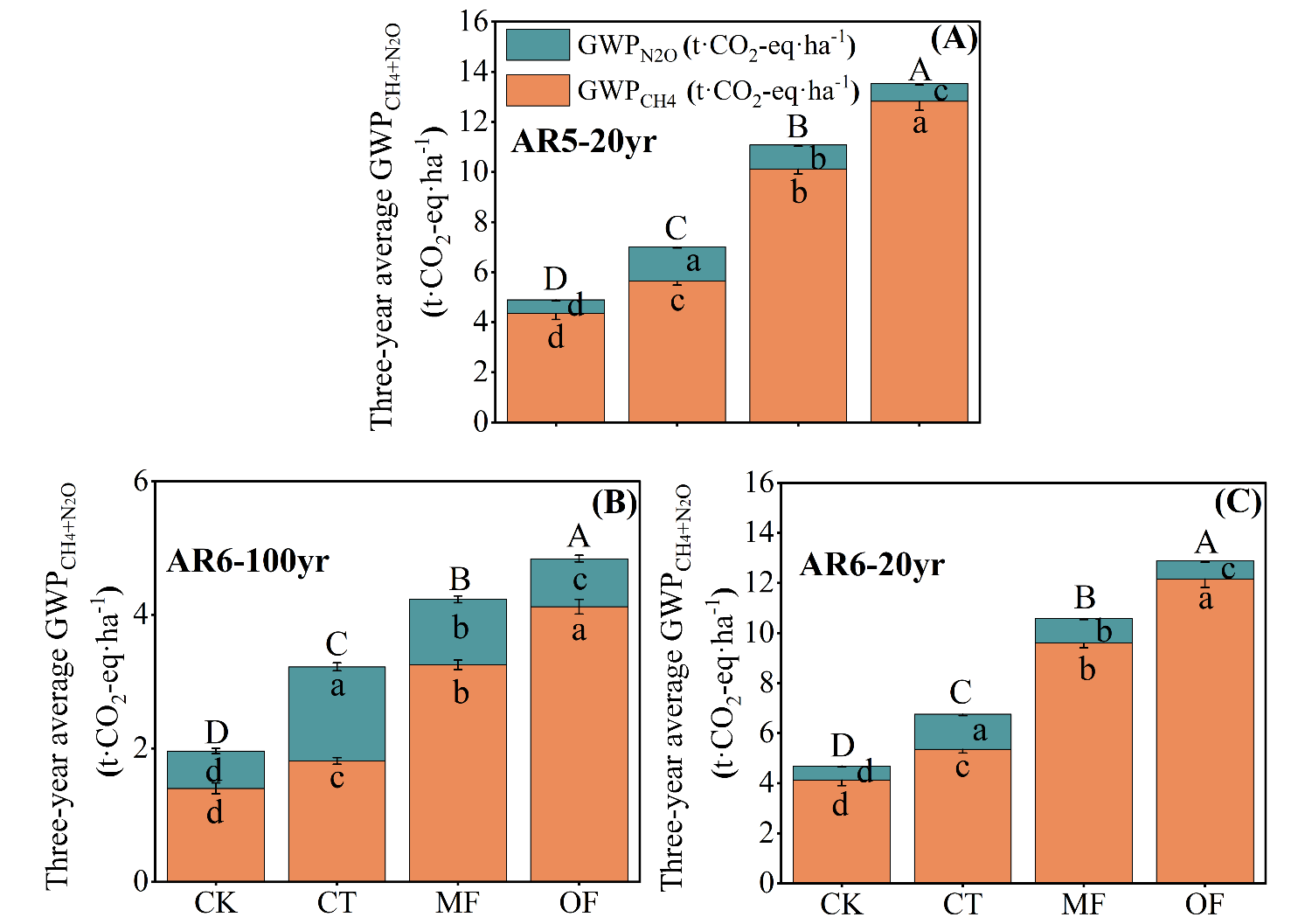


**Supplementary Figure 6.** Three-year average GWP during the rice growing season from 2023 to 2025 under different fertilization treatments (A: IPCC AR5 20-year time horizon, with CH₄ set at 84 and N₂O at 264; B: IPCC AR6 100-year time horizon, with CH₄ set at 27 and N₂O at 273; C: IPCC AR6 20-year time horizon, with CH₄ set at 79.7 and N₂O at 273). Lowercase letters indicate significant differences in the GWP of CH₄ and N₂O among treatments at the *p* < 0.05 level. Uppercase letters indicate significant differences in the total GWP of CH₄ and N₂O among treatments at the *p* < 0.05 level.


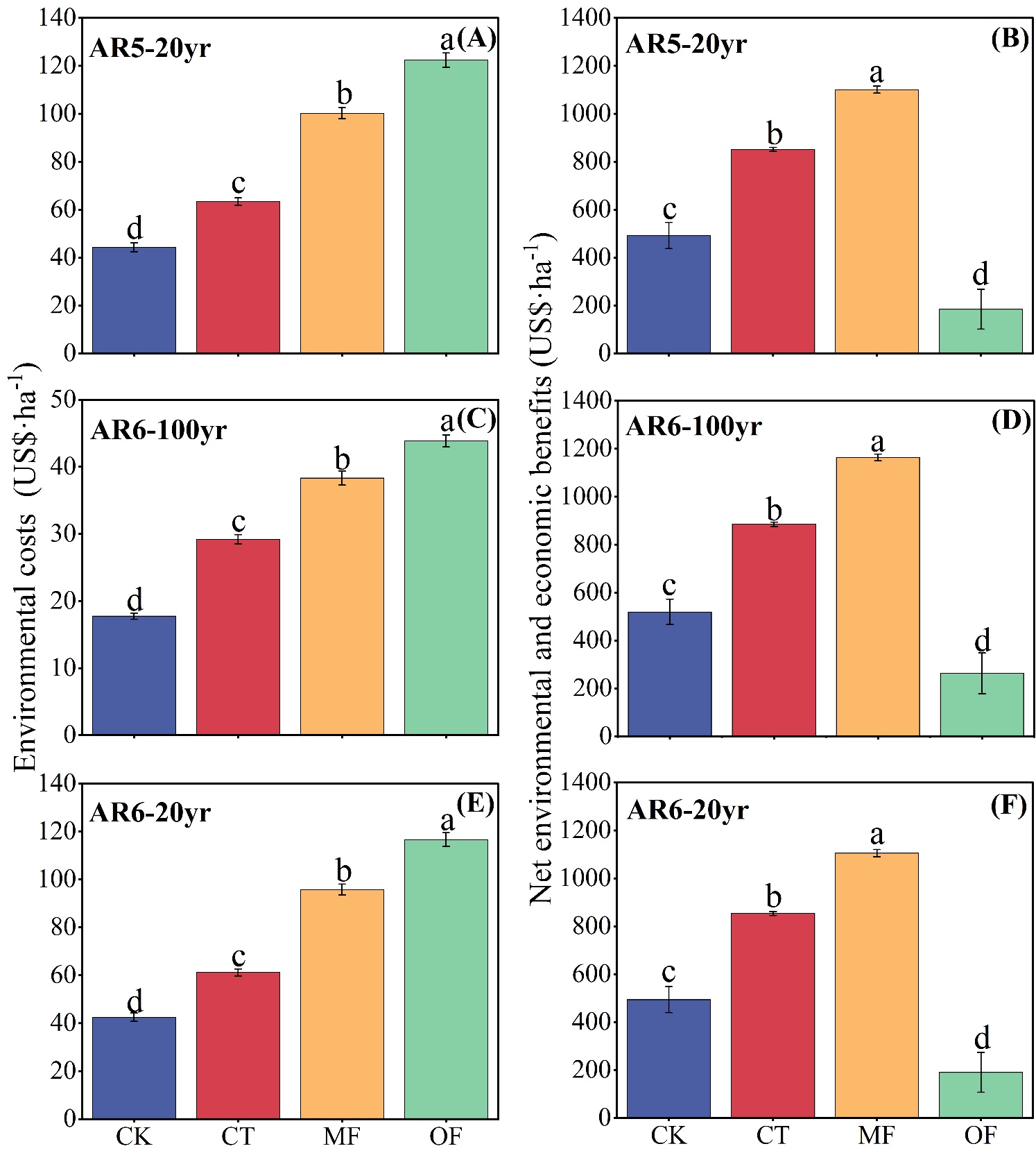


**Supplementary Figure 7.** Environmental costs (A, C, E) and NEEB (B, D, F) calculated under IPCC AR5-20 yr (A,B), AR6-100 yr (C,D) and AR6-20 yr (E,F) during rice growing seasons under different fertilization treatments from 2023 to 2025. Different lowercase letters indicate significant differences between treatment groups at the *p* < 0.05 level.

## Supplementary Tables

**Supplementary Table 1.** Detailed fertilizer management under different treatments in 2023 - 2025（kg N ha^-1^）

| Treatments | Basal  Fertilizer | | Tillering fertilizer | Booting fertilizer | Total nitrogen fertilizer application |
| --- | --- | --- | --- | --- | --- |
|  | Organic  N | Inorganic N | Inorganic N | Inorganic N |  |
| CK | - | - | - | - | - |
| CT | - | 180 | 60 | 60 | 300 |
| MF | 150 | 90 | 30 | 30 | 300 |
| OF | 300 | - | - | - | 300 |

**Supplementary Table 2.** Prices used in the cost-benefit analysis.

| Item | Price |
| --- | --- |
| Grain price (US$ kg^-1^) | 0.38 |
| Seed (US$ kg^-1^) | 107.10 |
| Farm machinery (US$ kg^-1^) | 597.80 |
| Synthetic N (US$ kg^-1^) | 627.40 |
| P_2_O_5_ (US$ kg^-1^) | 727.80 |
| K_2_O(US$ kg^-1^) | 533.90 |
| Pesticide (US$ kg^-1^) | 130.80 |
| Labor (US$ kg^-1^) | 1011.00 |
| Indirect cost (US$ kg^-1^) | 38.60 |
| Commercial manure (US$ kg^-1^) | 72.80 |

**Supplementary Table 3.** Main farming period in the experimental field.

| Rice season | Transplanting | Recovery stage | Tillering stage | Booting stage | Heading stage | Grain filling stage | Harvest |
| --- | --- | --- | --- | --- | --- | --- | --- |
| 2023 | 2023/6/16 | 2023/6/25 | 2023/7/9 | 2023/8/17 | 2023/9/6 | 2023/10/8 | 2023/11/18 |
| 2024 | 2024/6/12 | 2024/6/21 | 2024/7/18 | 2024/8/8 | 2024/9/13 | 2024/10/10 | 2024/11/14 |
| 2025 | 2025/6/10 | 2025/6/18 | 2025/7/10 | 2025/8/7 | 2025/9/11 | 2025/10/9 | 2025/11/21 |

Note：2023, 2024, and 2025 refer to the rice growing seasons of those years, respectively.

**Supplementary Table 4.** Rice yield and its composition under different fertilization treatments during 2023-2025.

|  | 2023 | | | 2024 | | | 2025 | | |
| --- | --- | --- | --- | --- | --- | --- | --- | --- | --- |
| Treatment | Panicle numbe  （*10^4^ ha^-1^） | grain number per spikes | 1000- grain weight (g) | Panicle number  （*10^4^ ha^-1^） | grain number per spikes | 1000-grain weight (g) | Panicle number  （*10^4^ ha^-1^） | grain number per spikes | 1000-grain weight (g) |
| CK | 292.50  ±0.5b | 103.00  ±6.33b | 20.19  ±1.17a | 264.33  ±19.97b | 116.75  ±8.75a | 25.68  ±0.38a | 343.5  ±10.68c | 88.17  ±6.19b | 26.66  ±1.01a |
| CT | 425.00  ±12a | 132.84  ±11.17a | 18.41  ±0.62a | 397.00  ±11.00a | 130.25  ±0.55a | 26.97  ±1.14a | 574.50  ±20.50a | 117.43  ±23.42ab | 26.01  ±1.16a |
| MF | 424.66  ±22.83a | 122.00  ±3.00ab | 19.34  ±0.38a | 322.50  ±2.5b | 128.50  ±1.50a | 26.52  ±1.76a | 474.67  ±10.73b | 151.00  ±26.17a | 25.83  ±1.23a |
| OF | 301.00  ±10.58b | 115.00  ±4.00ab | 19.82  ±1.27a | 303.20  ±18.75b | 123.00  ±11.50a | 25.73  ±0.25a | 389.00  ±19.05c | 93.73  ±1.59ab | 26.33  ±0.63a |

Different lowercase letters show significant difference among treatments (*p* < 0.05).
